# Hand Washing: When Ritual Behavior Protects! Obsessive–Compulsive Symptoms in Young People during the COVID-19 Pandemic: A Narrative Review

**DOI:** 10.3390/jcm11113191

**Published:** 2022-06-02

**Authors:** Francesco Demaria, Maria Pontillo, Cristina Di Vincenzo, Michelangelo Di Luzio, Stefano Vicari

**Affiliations:** 1 Child and Adolescence Neuropsychiatry Unit, Department of Neuroscience, IRCCS (Istituto di Ricovero e Cura a Carattere Scientifico) Bambino Gesù Children’s Hospital, 00165 Rome, Italy; maria.pontillo@opbg.net (M.P.); cristina.divincenzo@opbg.net (C.D.V.); michelangelo.diluzio@opbg.net (M.D.L.); stefano.vicari@opbg.net (S.V.); 2 Department of Life Sciences and Public Health, Università Cattolica del Sacro Cuore, 00168 Rome, Italy

**Keywords:** obsessive–compulsive symptoms, OCD, hand washing, young people, COVID-19, pandemic

## Abstract

The Coronavirus Disease 2019 (COVID-19) pandemic had a profound impact on the lifestyles and mental health of young people. It has been hypothesized that the focus on hygiene and the fear of contamination/infection during the pandemic may have exacerbated obsessive–compulsive (OC) symptoms in this population. OC symptoms are widespread in the general population, with varying degrees of intensity. At their most extreme, they manifest in obsessive–compulsive disorder (OCD), which is characterized by obsessive thoughts and compulsive behaviors. The present narrative review aimed at evaluating the relationship between the COVID-19 pandemic and OCD and OC symptoms in young people, especially children and adolescents with and without OCD, focusing on vulnerability and risk factors and the impact of lockdown measures. Of the six studies identified, four examined clinical samples diagnosed with OCD and two looked at community-based adolescent samples. Five of the six studies found that OC symptoms increased during the pandemic. Additionally, vulnerability to anxiety may constitute a risk condition and the lockdown measures and personal stressful life events can constitute potential triggers of OC symptoms, while ongoing treatment for OCD had a protective effect. The results suggest that, during the COVID-19 pandemic, obsessive and compulsive behavior (e.g., hand washing) in young people at the greatest risk should be monitored, and the intervention of mental health services should be maintained. More research is needed in this area.

## 1. Introduction

On 11 March 2020, the World Health Organization declared a COVID-19 pandemic [1]. To reduce the spread of the Severe Acute Respiratory Syndrome Coronavirus 2 (SARS-CoV-2 virus), extraordinary containment measures were adopted across the world, including lockdowns, social distancing, and protective individual measures. These measures brought about significant social changes that impacted the lifestyles and mental health of young people [2,3]. Racine et al. [4] estimated that, globally, during the first year of the pandemic, 25% of children and adolescents suffered from depression and 20% suffered from anxiety—rates that doubled those of the pre-pandemic era. Additionally, Raymond et al. [5] detected symptoms of post-traumatic stress and anxiety in young people with socio-emotional vulnerability during the pandemic, noting that females and adolescents showed the most symptoms, relative to males and children.

Pandemics and their associated containment measures represent psychosocial adversity factors that may significantly affect young people and their families [6]. In particular, research has shown that increased stress and anxiety during a pandemic may exacerbate symptoms of obsessive–compulsive disorder (OCD) in young people [7].

Indeed, some authors [8] have reported an association between increased diagnoses of obsessive–compulsive disorder (OCD) and certain COVID-19 factors, including lockdown measures, the focus on hygiene, and family adversities that intensified fear of contagion.

While obsessive–compulsive (OC) symptoms are widespread in the general population, when they present at a high level, they constitute OCD. OCD is a neuropsychiatric disorder characterized by intrusive, repetitive, and unwanted (i.e., obsessive) thoughts, accompanied by compulsive behaviors or mental acts [9]. Among children and adolescents, the disorder has an estimated global prevalence of 1–3% [10], similar to the rate observed in adults [11]. The most frequent comorbidities are anxiety (70%) and depressive (30%) disorders [9]. OCD can significantly compromise quality of life [12,13], and it manifests with different clinical subtypes (i.e., contamination/washing, checking, symmetry, forbidden thoughts). The most common [14] subtype involves an obsession with contamination/infection (e.g., with dirt and/or germs), which activates cleaning/washing compulsions (e.g., hand washing) to reduce anxiety. Avoidance behavior generally also arises in an attempt to prevent contamination, with the result that situations are avoided for fear of coming into contact with objects that could be “contaminated”.

The focus of the present study is to improve our understanding of OCD and to investigate the mechanisms of onset of OC symptoms, which are not easily recognizable in children and adolescents. Although OCD is considered an early onset neuropsychiatric disorder (i.e., 50%) of adults with OCD report that their OC symptoms began before the age of 18 [15]), and despite considerable research to date, OCD remains poorly understood. The literature on OCD during the COVID-19 pandemic is richer for the adult population than for the child and adolescent populations. In adults, a worsening of OC symptoms has been confirmed, in particular with respect to the contamination/washing subtype during the initial public health measures and restrictions [16]. In young people, worsening of the contamination/washing subtype has been shown to be less evident than a general worsening of OC symptoms. In both populations, vulnerability to stress has been found to be associated with a worsening of OC symptoms [17].

It has been hypothesized that the public health measures that were put into effect to manage and contain the spread of the SARS-CoV-2 virus (e.g., hand washing) and fear of contamination/infection may have increased the severity of OC symptoms in young people during the pandemic. Additionally, media coverage of the pandemic may have amplified perceptions of threat and increased levels of stress and anxiety, in relation to the number of infections, the spread of the virus, and the protection measures in place [18]. Studies on the adult population have shown that OC symptoms did indeed worsen during the pandemic. Alonso et al. [19], in a longitudinal study of adults with OCD (*N* = 127), found that 65.3% of patients reported a worsening of OC symptoms and washing compulsions. In addition, a study of the general population in Saudi Arabia (*N* = 2909) [20] found new-onset obsessions in 57.8% and new-onset compulsions in 45.9% of the participants, and significantly more obsessions with dirt, germs, and viruses; additionally, 72.4% of the participants reported moderate/high stress.

Some recent studies have reviewed the effects of the COVID-19 pandemic on OC symptoms [8,17,21]. Cunning and Hodes [8] studied the effect of the COVID-19 pandemic on OCD and OC symptoms in young people. Grant et al. [17] considered the OC symptoms of young and adult clinical and general populations in a rapid scoping study. Zaccari et al. [21] considered the impact of COVID-19 on OCD in child, adolescent, and adult clinical populations.

The present narrative review aimed at evaluating the relationship between the COVID-19 pandemic and OCD and OC symptoms in young people, especially children and adolescents with and without OCD, focusing on vulnerability and risk factors and the impact of lockdown measures.

## 2. Materials and Methods

The research drew on a selective review of the literature published between 1 January 2020 and 12 March 2022, following the guidelines of the Preferred Reporting Items for Systematic Reviews and Meta-Analyses (PRISMA).

### 2.1. Search Strategy

The literature review was based on a keyword search of the PubMed, CINAHL, PsycInfo, MedLine, and Cochrane Library electronic databases. The following keywords were used: (OCD OR compulsive OR obsessive–compulsive) and (COVID OR COVID-19). The search was conducted on 12 March 2022.

### 2.2. Inclusion and Exclusion Criteria

The included studies investigated the effect of the COVID-19 pandemic on OCD and OC symptoms in young people. Reviews, meta-analyses, comments, and letters were excluded. No language or study design restrictions were applied.

### 2.3. Selection Procedure, Data Extraction, and Data Management

**Figure 1:**
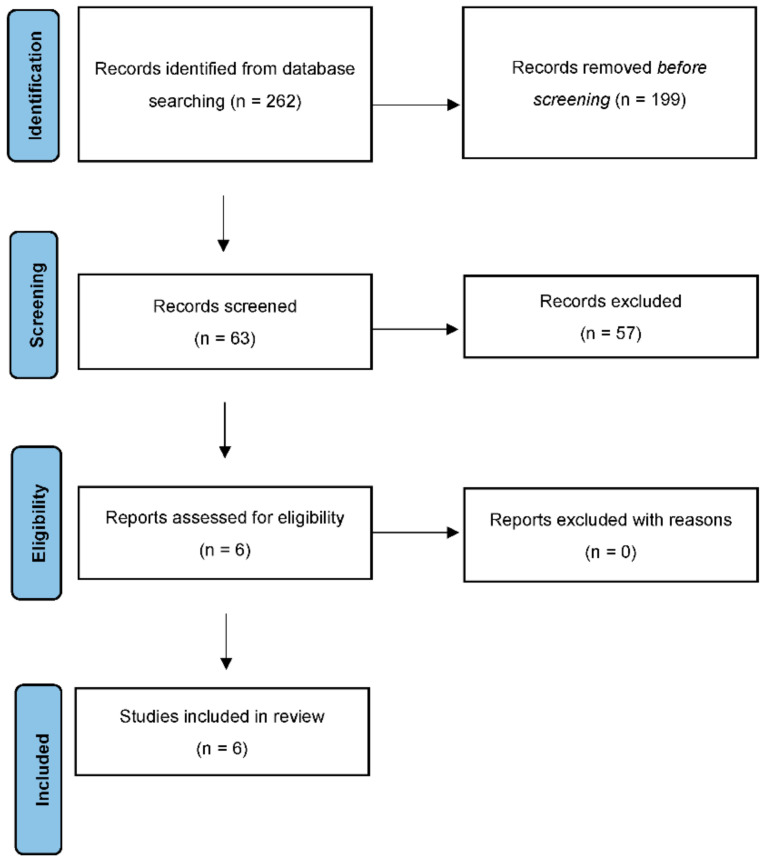
PRISMA 2009 Flow Diagram.

The bibliographies of the most important articles of interest were examined. Three authors (F.D., M.P., S.V.) independently extracted data on efficacy, acceptability, and tolerability. Other reviewers (C.D.V., M.D.L.) selected the final articles. Figure 1 presents a detailed flow chart of the study selection process.

The search algorithm retrieved 262 articles, of which 199 were excluded based on the abstract, because it was not in the field of our research.

Of the 63 studies screened, 57 were excluded for the reasons listed in Table 1.

A total of 6 articles were selected and included in this review.

In terms of evidence-based medicine, the quality of the included studies was moderate.

## 3. Results

Of the six selected papers, four reviewed clinical populations (including adolescents with OCD in one paper) and two examined general populations of young people. The studies were heterogeneous in terms of their characteristics and tools, and they each applied blocking measures of varying levels of severity (Table 2). Of the four clinical population studies, three found a significant increase in OC symptoms during the pandemic.

In detail, Tanir et al.’s [75] longitudinal study collected data through telephone and online interviews with patients and their parents. Patients’ CY-BOCS scores 6 months prior to the first confirmed case of COVID-19 in Turkey were collected as pre-pandemic data. The results showed a worsening of OC symptoms in 54.09% of the sample (*n* = 33/61) during the pandemic, with 36% showing at least a 30% increase in their CY-BOCS score, 34% reporting no change in symptom severity, and only 11% reporting a decrease in their CY-BOCS score (mean CY-BOCS scores were 14.24 ± 5.05 and 19.0 ± 6.89 before and during the pandemic, respectively). The authors also found a significant increase in the frequency of contamination obsessions (*p* = 0.008) and cleaning/washing compulsions (*p* = 0.039). Contamination obsessions and cleaning/washing compulsions were the most frequently reported symptoms, both before and during the pandemic. Only 11.4% of the adolescent patients had the support of cognitive–behavioral psychological therapy during the pandemic. The authors hypothesized that the continuous media information on the possible health consequences related to COVID-19 infection and COVID-19 protective measures increased perceived threat and responsibility in young people with OCD.

Nissen et al.’s [76] cross-sectional study administered a questionnaire to a clinical group and a survey group. The clinical group (CG) consisted of children and adolescents (*n* = 65) who had recently been diagnosed with OCD in a specialized clinic, and were receiving psychological and/or psychiatric treatment. The survey group (SG) was comprised of adolescents (*n* = 37) who had been diagnosed with OCD years ago and were not currently in treatment. During the pandemic, the SG reported a more significant increase in OC symptoms (77%) relative to the CG (44.6%). Ten subjects (15.4%) in the CG experienced new OC symptoms related to thoughts about COVID-19, which became integral to their OCD profile. Thoughts about COVID-19 were also positively correlated with aggravation of OCD during the pandemic. Obsessions with aggressive and sexual content, combined with a lack of insight, predicted a worsening of OC symptoms (*p* = 0.02). Furthermore, aggravation of OCD was correlated with increased anxiety, depressive symptoms, and avoidance behavior. The SG presented the most significant increase in symptoms across all parameters. The authors equated fear of COVID-19 with a traumatic experience capable of exacerbating psychological disorders and activating or worsening OC symptoms. They hypothesized that, during the pandemic, their young participants may have experienced trauma related to serious illness (or even death) within the family. Of note, a limitation of the study is that the groups significantly differed in size (SG: 37/600 = 6% vs. CG: 65/101 = 64%).

Khan et al.’s [78] exploratory, cross-sectional study considered adolescents with different mental disorders (i.e., neurodevelopmental, disruptive, impulse control, and conduct disorders; mood and anxiety disorders; OCD) at the Community Child and Adolescent Mental Health Service (CAMHS) at Hamad Medical Corporation in Qatar. CAMHS is Qatar’s only community-based medical service for children and young people below the age of 18 years with moderate to severe mental and behavioral disorders. Pearson’s correlation coefficient (*r*) was used to explore the relationship between fear of COVID-19 and the development of OC symptoms. Out of 63 patients, 57 (90.4%) scored 12 or higher on the COVID-19 Inventory, suggesting significant pandemic-related worry; and 31 had an OCI-R modified score of 17 or higher, indicating significant OC symptoms during the pandemic. A positive and significant correlation was found between these scores (*r* = 0.405, sig. [two-tailed] = 0.001). Illustrating this significant relationship, seven out of eight patients scored above the OCI-R cut-off and scored 12 or higher on the COVID-19 Inventory.

Schwartz-Lifshitz et al. [77] conducted a longitudinal study during the first wave of the COVID-19 pandemic (*N* = 29). The study included children and adolescents from a large tertiary hospital with a primary diagnosis of OCD. The CGI-S was applied twice: once during the pandemic and a second time at a follow-up. A two proportion *Z*-test showed that a higher proportion of children and adolescents with OCD improved during the pandemic than deteriorated (*Z* = 2.23, *p* = 0.02). Mean OCI-CV scores were low–medium (mean = 12.75, SD = 7.66). Based on the CGI-S, the majority of patients (*n* = 16, 55%) reported improvement (mean = 4.83, SD = 1.53). Furthermore, a two proportion *Z*-test based on a self-report functioning questionnaire showed that a higher proportion of children with OCD reported improved functioning than deteriorated functioning (*Z* = 4.20, *p* < 0.0001). The authors noted that immediate intervention by a psychiatric service allowed 12 participants (42%) to receive psychotherapeutic treatment online during the pandemic, ensuring greater containment of anxiety and stress. All participants were receiving psychiatric and/or psychotherapeutic treatment at follow-up.

Two studies examined the general population of young people. Secer et al.’s [79] cross-sectional study considered 598 high school students, who were recruited using a convenience sampling method. A link to an online survey was emailed or texted to students. The survey was designed to evaluate the relationship between fear of COVID-19 and OCD, considering the mediating role of emotional reactivity, experiential avoidance, and anxiety–depression. The results demonstrated that fear of COVID-19 positively predicted emotional reactivity (β = 0.50, *p* < 0.01), and emotional reactivity positively predicted experiential avoidance (β = 0.59, *p* < 0.01) and depression–anxiety (β = 0.81, *p* < 0.01). Furthermore, experiential avoidance had a positive and significant predictive effect on OCD (β = 0.12, *p* < 0.01) and depression–anxiety on OCD (β = 0.82, *p* < 0.01). The results suggested that fear of COVID-19 was a strong predictor of OCD. Emotional reactivity and experiential avoidance also increased the risk of psychosocial disorder.

Darvishi et al. [80] considered 150 high school and pre-university students, who were randomly sampled in a cross-sectional study. The authors found that 67.3% demonstrated OC symptomatology. The prevalence of OC symptoms was slightly higher in women than in men (72.1% vs. 60.3%). Similarly, there was a significant difference in rates of obsession between genders (*p* = 0.001). The descriptive findings showed that washing compulsions were the most common OC symptom. The study applied the Maudsley Obsessive–Compulsive Inventory, which is not a diagnostic tool, but is used to screen OC symptoms in clinical and non-clinical populations. The authors noted that the COVID-19 pandemic may have particularly affected young people at greatest risk of developing OCD. Specifically, these youths may have developed compulsive hand washing behaviors when they learned that washing, which was strongly recommended by public health authorities, relieved their anxiety. These behaviors may have been attributable to a fear of contagion.

## 4. Discussion

The COVID-19 pandemic is likely to have exacerbated OC symptoms in young people, as all but one [77] of the investigated studies detected a significant increase in OC symptoms during the pandemic. In Schwartz-Lifshitz et al.’s [77] study, the mean OCI-CV scores were low–medium (mean = 12.75, SD = 7.66) and the majority of patients (55%) reported an improvement in OC symptoms (mean = 4.83, SD = 1.53). However, the contradictory results may be due to the small sample size (*N* = 29). Additionally, the recruitment period (1 April 2019 to 31 March 2020) was significantly longer than that of the other studies.

The worsening OC symptoms reported in both the clinical and the general populations in the investigated studies led us to reflect on the impact of the COVID-19 pandemic on all young people, indiscriminately. The study by Nissen et al. [76] noted an increase in the severity of OC symptoms in both groups (CG = 73%; SG = 44.6%). In Khan et al.’s [78] sample, patients with different neurodevelopmental disorders (including seven of the eight patients diagnosed with OCD) showed a statistically significant relationship between worry about the pandemic and OC symptoms. In Darvishi et al. [80], 67.3% of the general population sample of students demonstrated OC symptoms, with washing compulsions the most prevalent. Finally, 54.09% of Tanir et al.’s [75] clinical sample reported a worsening of OC symptoms, with a significant increase in the frequency of contamination obsessions (*p* = 0.008) and cleaning/washing compulsions (*p* = 0.039).

All of these studies were based in countries where lockdown measures were in place to manage the spread of the SARS-CoV-2 virus. While reduced contact with the outside world can protect against external stressors and thereby potentially alleviate OC symptoms, the sudden change in daily routines may also provoke anxious and maladaptive reactions that can trigger OC symptoms. In particular, the interruption of school and sports activities, as well as the limitation of social relations, can create unfavorable conditions for the psychophysical well-being of young people [81], increasing their anxiety, depression, and avoidance behaviors, and thereby aggravating their functioning and increasing their risk of manifesting OC symptoms [76,79]. Indeed, Secer et al. [79] showed that the effect of fear of COVID-19 on OCD may be mediated by emotional reactivity, experiential avoidance, and anxiety–depression, and that avoidance and depression–anxiety significantly predict OCD.

In children and adolescents, OC symptoms have an insidious onset, often exacerbated by stressful life events. Vulnerability to anxiety or the presence of a frank comorbid anxiety disorder (frequent in OCD) may therefore constitute a risk condition. Anxiety disorder shares features and clinical manifestations with OCD, including overthinking, excessive worry, and avoidance. The impact on overthinking and on excessive worry was exceptionally strong in the first wave of the pandemic. An unknown virus, the absence of treatments, and unanswered questions can stimulate automatic thoughts on contagion and disease in the anxious subject with possible trigger or increase in OC symptoms. In the works considered [76,78,79], worsening of OC symptoms was concomitant with an increase in the level of anxiety caused by stress and fear of the pandemic. In Darvishi et al. [80], young people were found to engage in compulsive hand washing to relieve anxiety and reduce fear/obsession over contagion. Additionally, lockdown measures at the start of the pandemic played an absolutely unfavorable role, representing a further potential trigger of OC symptoms and OCD. Children and adolescents have experienced radical changes and out of control situations that have increased stress and fear levels. Indeed, in our review, all studies that found a worsening of OC symptoms were based on data from March/April 2020, when uncertainty was extremely high. This could be in line with studies that proposed that the role of intolerance of uncertainty could be critical in increasing anxiety and OC symptoms [82,83]. Improvement in OC symptoms and OCD, proposed by Schwartz-Lifshitz et al. [77], could be due to immediate online psychotherapeutic intervention. Indeed, online psychological support focusing on containing anxiety and stress could be useful to improve OC symptoms and OCD. Finally, after the first phase of the COVID-19 pandemic, a reduction in stress and fear of contagion may be hypothesized in relation to individual factors (e.g., coping strategies) and environmental factors (e.g., partial reduction in lockdown measures). However, these findings could be considered with caution because self-report data of studies included might generate a significant bias when pre and peripandemic data are compared.

A lockdown is likely to be stressful for the entire family. In this situation, parents may find it difficult to support a child with OCD and to avoid personal involvement in OC rituals and anxiety. In fact, Albert et al. [84] found that 80–90% of families with an OCD child directly participate in their child’s symptoms through family accommodation behaviors.

It is crucial that mental health services ensure adequate psychological/psychiatric care when pandemic-related restrictions are in place. Tanir et al. [75] found that only 11.4% of their adolescent participants had psychological support during the COVID-19 pandemic. Additionally, 73% of Nissen et al.’s [76] SG, who did not have immediate contact with psychiatric services during the pandemic, reported more intense OC symptoms. Schwartz-Lifshitz et al. [77] found that 42% of their adolescent participants (i.e., 12 out of 29) benefited from online psychotherapeutic treatment during the pandemic.

Of concern, a WHO survey conducted from June to August 2020 in 130 countries found a 72% disruption in mental health services for children and adolescents due to COVID-19 [85]. If children and adolescents are not adequately supported during times of stress, external situations may precipitate the development or worsening of psychopathological conditions. In particular, young people with OCD who manifest intense distress, poor insight, and unusual OC symptoms may be at risk of developing more serious psychopathology, such as psychosis risk syndrome [86].

Pandemics and their associated containment measures may be experienced as traumatic [6]. Indeed, Nissen et al. [87] equated fear of COVID-19 with a traumatic experience that could exacerbate psychological disorders and activate OC symptoms. Furthermore, Dykshoorn [33] found a significant correlation between OCD and traumatic events. Accordingly, trauma should be considered in any discussion of OC symptoms. Intrusive thoughts in response to a traumatic event can eventually structure themselves as obsessive thoughts (e.g., of contamination) and, as in OCD, manifest repetitive behaviors (e.g., washing compulsions) to counteract the distressing obsessions. The specific characteristics of the traumatic event may play a role in determining the OCD symptom profile [88].

If a pandemic is experienced as a traumatic event (or even just a stressful life event), it could trigger OC symptoms. More specifically, fear of contagion, protective measures, media coverage, environmental factors, individual susceptibility, and personal life trauma may represent stress and anxiety inducing factors that could trigger OC symptoms in young people. Understanding how these factors might exacerbate OC symptoms is important not only to improve diagnostic and therapeutic tools, but also to predict the pathogenesis and evolutionary trajectory of the disorder. Child and adolescent mental health services must ensure timely interventions during exceptional times (e.g., a pandemic involving a lockdown or social restrictions). In these periods, alternative intervention strategies should be considered.

Alternative intervention strategies refer to digital psychiatry, which may include artificial intelligence, telepsychiatry, and a host of internet-based computer-assisted technologies, used remotely in the service of mental health. In particular, these new technologies could be very useful for high-risk groups such as confirmed coronavirus patients, quarantined people and those with vulnerable psychological traits [89].

Some limitations should be considered in our review. Firstly, there is a discordance of study design and measures for clinical assessment between the included studies. In addition, they examined a small sample of young people with OCD. This does not allow a quantitative analysis of the results and limits the interpretability of the findings.

## 5. Conclusions

The review found that the COVID-19 pandemic was likely to have exacerbated OC symptoms in young people. The investigated studies were conducted in countries where lockdown measures were in place to manage the spread of SARS-CoV-2. Vulnerability to anxiety may constitute a risk condition and the impact of lockdown measures and personal stressful life events may constitute a potential trigger for OC symptoms. It is imperative that mental health services maintain adequate psychological/psychiatric care considering alternative intervention strategies when pandemic-related restrictions are in place. More research is needed in this area, especially with long-term follow-up, large samples, and structured interviews.

## Figures and Tables

**Table 1:** List of excluded studies along with reasons for exclusion.

Reason for Exclusion	Study Name
Article format (review, conference proceedings, comments, editorials or letters)	Aardema, F. [22]; Banerjee, D.D. et al. [23]; Dennis, D. et al. [24]; Fineberg, N.A. et al. [25]; Fontenelle, L.F. [26]; Jassi, A. et al. [27]; Kumar, A. et al. [28]; Guzick, A.G. et al. [16]; Liu, W. et al. [29]; Ornell, F. et al. [30]; Sheu, J.C. et al. [31]; Sulaimani, M.F. et al. [32]; Uzunova, G. [33]; Zaccari, V. et al. [21].
Characteristics of sample: only adult participants included	Abba-Aji, A. et al. [34]; Acenowr, C.P. et al. [35]; Alateeq, D.A. et al. [20]; Alhujaili, N. et al. [36]; Loosen, A.M. et al. [37]; Alonso, P. et al. [19]; Tandt, H.L. et al. [38]; Benatti, B. et al. [39]; Chakraborty, A. et al. [40]; Davide, P. et al. [41]; Fang, A. et al. [42]; Fontenelle, L.F. et al. [43]; Hassoulas, A. et al. [44]; Højgaard, D.R.M.A. et al. [45]; Jelinek, L. et al. [46]; Jelinek, L. et al. [47]; Jelinek, L. et al. [48]; Ji, G. et al. [49]; Khosravani, V. et al. [50]; Khosravani, V. et al. [51]; Liao, J. et al. [52]; Meșterelu, I. et al. [53]; Rosa-Alcázar, Á. et al. [54]; Rosa-Alcázar, Á. et al. [55]; Pinciotti, C.M. et al. [56]; Rivera, R.M. et al. [57]; Samuels, J. et al. [58]; Sharma, L.P. et al. [59]; Tandt, H.L.N. et al. [60]; Wheaton, M.G. et al. [61]; Wheaton, M.G. et al. [62]; Zaccari, V.et al. [63]; Zheng, Y. et al. [64].
Study design: case report or case series, intervention study	Alkhamees, A.A. [65]; Carmi, L. et al. [66]; French, I. et al. [67]; Hosseini, S.V. et al. [68]; Jain, A. et al. [69]; Jansen, T. et al. [70]; Kumar, P. et al. [71]; Sejdiu, A.; et al. [72]; Sowmya, A.V. et al. [73]; Uvais, N.A. et al. [74]

**Table 2:** Study findings of the effect of COVID-19 on OCD and OC symptoms in young people.

Author (Year)	Location	Study Design, Sample Size and Age Range	Outcome Measurement Method/Time of Data Collection	Results
Tanir et al. [75]	Turkey	Longitudinal study, 61 patients with OCD Aged 6–18 years	CY-BOCS and CGI-S/pre-pandemic (September 2019–March 2020) and during the pandemic (April 2020)	OCD symptom severity increased in 54% of patients during the pandemic. There were significant increases in contamination obsessions (*p* = 0.008) and cleaning/washing compulsions (*p* = 0.039) during the pandemic.
Nissen et al. [76]	Denmark	Cross-sectional study, 102 patients with OCD Aged 7–21 years	Self-report questionnaire based on the CY-BOCS used to measure change in OCD severity (on a Likert scale) (April–May 2020)	Both study samples reported an increase in OCD severity during the pandemic: 73% SG and 44.6% CG.
Schwartz-Lifshitz et al. [77]	Israel	Longitudinal study, 29 patients with OCD Aged 14–19 years	CGI-S, CGI-I, OCI-CV, self-report functioning questionnaire (scored on a scale ranging from 1 [very much improved] to 7 [very much worsened]/pre-pandemic (April 2019–March 2020) and during the pandemic (April–May 2020)	Mean OCI-CV scores were low–medium (mean = 12.75, SD = 7.66). Based on the CGI-S, the majority of patients (55%) reported symptom improvement during the pandemic (mean = 4.83, SD = 1.53).
Khan et al. [78]	Qatar	Cross-sectional study, 63 patients with developmental disorders Aged 14–18 years	COVID-19 Inventory (adapted from the Swine Flu Inventory, which is a pool of 10 items; a cut-off score of 12 was considered clinically significant for COVID-19 fear), OCI-R/pre-pandemic (July 2019–December 2019)	In total, seven out of eight patients with OCD reported a significant association between COVID-19 fear and obsessive-compulsive symptoms.
Secer and Ulas et al. [79]	Turkey	Cross-sectional study, 598 students Aged 14–18 years	OCI-CV, Emotional Reactivity Scale, Depression and Anxiety Scale for Children, Fear of COVID-19 Scale COVID-19, experiential avoidance questionnaire/unspecified	Fear of COVID-19 was a strong predictor of OCD.
Darvishi E. et al. [80]	Iran	Cross-sectional study, 150 students Aged 14–19 years	MOCI, CEQ/unspecified	In total, 67.3% of students demonstrated OC symptoms. Washing compulsions were the most prevalent OC symptom.

CGI-S: Clinical Global Impression–Symptom Severity Scale; CGI-I: Clinical Global Impression–Improvement Scale; OCI-CV: Obsessive–Compulsive Inventory–Child Version; OCI-R: Obsessive–Compulsive Inventory–Revised; MOCI: Maudsley Obsessive–Compulsive Inventory Questionnaire; CEQ: Cognitive Errors Questionnaire; CG: clinical group; SG: survey group; OCD: obsessive–compulsive disorder; CY-BOCS: Children’s Yale–Brown Obsessive–Compulsive Scale.

## Data Availability

Not applicable.
